# A Computational Approach to Repurposing Natural Products for DprE1 Inhibition

**DOI:** 10.1155/sci5/2105236

**Published:** 2025-07-09

**Authors:** A. V. Snehalatha, N. V. Anil Kumar

**Affiliations:** Department of Chemistry, Manipal Institute of Technology, Manipal Academy of Higher Education, Manipal, Udupi 576104, Karnataka, India

**Keywords:** DprE1, MMGBSA, molecular docking, molecular dynamics, natural products

## Abstract

This study aimed to investigate the potential of natural products (NPs) as inhibitors of decaprenylphosphoryl-D-ribose 2′-epimerase (DprE1), an enzyme crucial in *Mycobacterium tuberculosis* cell wall synthesis. Over 100 NPs were screened for anti-TB properties. Subsequently, the binding mechanism of the most potent inhibitor to DprE1 was investigated using computational methods, including molecular docking and simulations. Three compounds (CNP0123918, CNP0041612, and CNP0281145) were identified with promising binding interactions within DprE1's active site. CNP0123918 emerged as the top candidate, exhibiting good interaction with key residues in DprE1. This study suggests that computer-aided drug repurposing holds potential as a successful strategy for identifying novel anti-TB drugs. These findings contribute to the development of novel DprE1 inhibitors. Future research will focus on in vitro assays and in vivo and toxicology assessment of CNP0123908 to establish its potential as an effective DprE1 inhibitor.

## 1. Introduction

*Mycobacterium tuberculosis* (MTB) is the causative agent of tuberculosis (TB), a highly contagious infectious disease. It is the leading cause of death from a single infectious agent and ranks in the top 10 causes of death globally. In 2022, an estimated 7.5 million people became ill with TB, and 1.3 million people died from the disease [1]. The majority of the world's TB cases are concentrated in just a few countries, India, Indonesia, Nigeria, Pakistan, the Philippines, Bangladesh, the Democratic Republic of the Congo, China, South Africa, and Kenya [2]. The rise in TB cases can be attributed to a number of factors, including poverty and malnutrition, urbanization, HIV/AIDS, and drug-resistant TB. Several actions can be taken to lessen the TB burden, such as funding TB preventive and control programs, dealing with the socioeconomic factors that contribute to the disease, and researching and developing novel TB treatments and diagnostics.

Many new pharmacological targets have been identified in the last 15 to 20 years as part of the quest for safe and effective anti-TB medicines [3, 4]. Unlike some other targets within bacteria, the cell wall is necessary for MTB survival and growth. Disruption of its integrity ultimately leads to bacterial death, making it a highly attractive target for therapeutic intervention [5]. DprE1 is a key enzyme in arabinogalactan biosynthesis and is essential for MTB cell wall integrity and growth. Inhibiting DprE1 disrupts cell wall synthesis, leading to bacterial death [6]. DprE1 works in tandem with DprE2 [7] as a part of a two-enzyme system responsible for converting decaprenylphosphoryl-β-D-ribose (DPR) into decaprenylphosphoryl-β-D-arabinose (DPA) [8]. DprE1 oxidizes DPR to form an intermediate DPX, and DprE2 reduces DPX to produce DPA. DPA is a crucial precursor for the biosynthesis of arabinogalactan and lipoarabinomannan, major components responsible for the synthesis of the mycobacterial cell wall [9].

New anti-TB medications have found DprE1 to be a suitable target [10]. There are numerous identified DprE1 inhibitors currently, including synthetic and natural compounds. New DprE1 inhibitors from natural products are reported [11]. They are safer and less problematic than man-made pharmaceuticals. Coumarins, terpenes, and flavonoids are some of the natural products that have been reported against the DprE1 enzyme [11–13]. The docking score of −8.935 kcal/mol against the DprE1 enzyme and a low IC_50_ value of 1.93 g/mL for biscoumarin with triazoles were reported by Khare et al. [14]. Recently, pyrazole-tethered coumarin compounds were reported by Kumar et al. [15] against DprE1 with a MIC of 6.25 g/mL and a binding affinity of −8.992 kcal/mol.

The traditional approach to designing a drug has a low success rate due to the high cost and time-consuming process. Thus, several chemoinformatic methods (molecular docking, virtual screening, and high-throughput screening (HTS)) have been employed to find pharmacophores that could target DprE1. A vast structure library can be virtually filtered through using similarity search, hierarchical docking, or pharmacophore models to identify potential drugs with the appropriate pharmacokinetic and dynamic characteristics [16]. Drug repurposing and repositioning strategies aid in the search for new uses for medications that have already undergone clinical testing and approval for another condition. Due to the availability of prior clinical safety data, it requires less time, costs less, and has a lower chance of failure [17].

**Figure 1:**
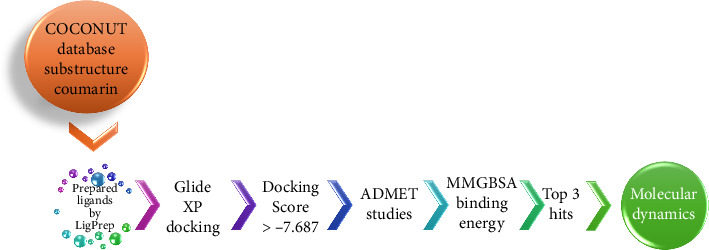
Workflow of the virtual screening process.

Historically, NPs have played a key role in drug discovery, especially for cancer and infectious diseases [18]. In this article, a drug repurposing technique and a structure-based virtual screening procedure were used to find NPs that were effective against the DprE1 enzyme. NPs in the COCONUT database were analyzed. Adverse drug reaction (ADME) studies, molecular mechanics generalized born surface area (MMGBSA) analysis, and Glide docking were used to further optimize the highest-scoring compounds. Studies using MD simulations that replicate the complex biological setting were used to confirm the outcomes of the molecular docking and MMGBSA analysis.  Figure 1 depicts the whole process flow of the virtual screening analysis. The results of this study may give a starting point for the optimization and development of novel DprE1 inhibitors to combat various strains of TB.

## 2. Materials and Methods

All the computational studies were carried out in Schrodinger Maestro Version 13.3.121, MMShare Version 5.9.121, Release 2023-4, by using ligand preparation, protein preparation, receptor grid generation, ligand docking, ADMET analysis, and MMGBSA analysis.

### 2.1. Database Preparation

COCONUT is one of the biggest and most well-annotated databases of natural items that is freely accessible to anyone. About four million unique NPs are available in the database [19]. Six hundred and twenty-six coumarin substructure NPs were downloaded from the database.

### 2.2. Ligand Preparation

To prepare the downloaded data for usage, LigPrep in the Schrodinger Suite [20] was used. Multiple conformations of each ligand can be constructed by considering the ring's flexibility and torsional angles, and it can be used to compute ligand attributes including ionization state, tautomers, and partial charges. One can filter the pool of possible ligands by size, polarity, and functional groups. From LigPrep, a total of 1967 ligands were obtained.

### 2.3. Protein Preparation

The RCSB protein data bank has the 1.79-Å resolution crystal structure of *MTB* DprE1 in combination with QN118 (PDB ID: 4P8N) [21]. The Schrodinger Suite's protein preparation wizard was utilized to get the protein ready, which removes all the water and unwanted chains, strengthens the H bonds, and adds any missing side chains. Crystal structure of the DprE1 was preprocessed, refined, and performed an energy minimization by using OPLS4 force field. By selecting the receptor grid creation option, a receptor grid box was generated, with the cocrystal ligand serving as the grid's centroid, allowing the protein's binding site to be defined.

### 2.4. Glide Docking

Ligand binding mode and affinity can be predicted via Glide XP docking [22, 23]. Multiple scoring systems and sampling strategies are employed to generate a large number of potential binding poses, which are then ranked according to their expected binding affinity. Glide's enhanced accuracy was used to dock the ligands obtained in LigPrep onto the premade protein.

### 2.5. ADMET Studies

Preclinical ADMET studies (absorption = the process by which a drug enters the bloodstream and is transported to various tissues and organs; distribution = the process by which a drug reaches its target tissues and organs; metabolism = the process by which a drug is transformed by enzymes in the body; excretion = the process by which a drug and its metabolites are eliminated from the body) are a power source of in vitro and in vivo tests used to evaluate the pharmaco drug candidates that are unlikely to succeed in clinical development due to poor absorption, distribution, metabolism, excretion, or toxicity and can be weeded out with the use of ADMET tests. Certain physicochemical properties were prioritized in ADME studies, such as Lipinski's rule of five (Ro5) because they strongly influence the compound's behavior in the body. (1) Molecular weight (Mol. wt ≤ 500 Pa): Smaller molecules tend to pass through biological membranes more easily via passive diffusion. High Mol. wt can hinder distribution, cell membrane permeability, and have poor absorption. (2) LogP (≤ 5): Moderate lipophilicity improves membrane permeability. Too high or too low values may lead to poor penetration, solubility, increased metabolism, and toxicity. (3) Hydrogen Bond Donors (HBD ≤ 5): More donor bonds lead to the formation of strong interactions with water and reduce membrane permeability. Too many donors lead to poor absorption because of high polarity. (4) Hydrogen Bond Acceptors (HBA ≤ 10): Like donors, too many acceptors lead to high polarity and reduce passive absorption [24]. These characteristics of the potential medicine can be predicted using Qikprop in the Schrodinger Suite [25].

### 2.6. Binding Free Energy by Using MMGBSA

Since entropy factor and solvation energy are difficult to compute using empirical scoring methods, they are not typically considered in glide docking. Therefore, the implicit solvation model was used to estimate the binding free energy value of the ligand–protein complex, which was found to be better in accuracy compared to the docking score. One typical method for determining the solvation free energy employs implicit water molecules and is known as MMGBSA [26]. The structure of the docked protein–ligand (PL) complex can then be imported into Maestro. The ligand's binding free energy will be determined. Stronger binding is indicated by a lower binding free energy.

MMGBSA is based on the following equation [27]:
  (1) $$\begin{array}{c} ∆G_{\text{binding}}=∆E_{\text{MM}}+∆G_{\text{solvation}}-T∆S. \end{array}$$
∆E_MM_ = molecular mechanics energy
• ∆E_MM_ includes all the internal energies (∆E_int_), electrostatic energy (∆E_ele_), and Van der Waals energies (∆E_vdW_)
• ∆E_MM_ = ∆E_int_ + ∆E_ele_ + ∆E_vdW_
∆G_Solvation_ = Solvation-free energy
• ∆G_Solvation_ term corresponds to the sum of a polar contribution (∆G_GB_) and a nonpolar contribution (∆G_SA_)
• ∆G_Solvation_ = (∆G_GB_) + (∆G_SA_)
T∆S = conformational entropy changes during complex formation

### 2.7. Molecular Dynamics

Desmond molecular dynamics is a useful technique for understanding the dynamic behavior of molecules [28]. It is utilized by scientists studying a wide range of topics, and it could revolutionize our knowledge of anything from proteins and nucleic acids to membranes and materials. A 10 × 10 × 10-Å orthorhombic box was used as the docking geometry for the ligand–protein combination. To neutralize the system, 0.15 M of Na^+^ and Cl^−^ were added, and the water molecules in TIP3P were employed as the solvent. OPLS4 was used as the underlying force field in this simulation. As part of the normal equilibration procedure, the system is typically reduced and pre-equilibrated before a production run. A manufacturing simulation lasting 200 ns was conducted at 1.01325 bar pressure and 300 K temperature, both within the normal pressure and temperature (NPT) ensemble. The simulation trajectory was analyzed, and the complex's stability was discussed by using root mean square deviation (RMSD), root mean square fluctuation (RMSF), and PL contacts.

RMSD: To measure the average change in displacement of atoms in a particular frame with respect to a reference frame, the RMSD is used. It is computed for every frame in the trajectory. RMSD for frame *x* is calculated using the following formula:

(2) $$\begin{array}{c} {\text{RMSD}}_{x}=\sqrt{\frac{1}{N}\overset{N}{\underset{i=1}{\sum }}{\left(r_{i}^{\prime }\left(t_{x}\right)-r_{i}\left(t_{\text{ref}}\right)\right)}^{2},} \end{array}$$

where *r*′ shows the position of selected atoms of particular frame (frame *x*) by superimposing with the reference frame, *t*_ref_ denotes the reference time (commonly the first frame is used as a reference and is considered at *t* = 0), *t*_*x*_ denotes the recorded time for frame *x*, and number of atoms is dented by *N*. This process is performed for all the frames along the simulation's trajectory.

RMSF: The RMSF is useful for characterizing local changes along the protein chain. The RMSF for residue *i* is

(3) $$\begin{array}{c} {\text{RMSF}}_{i}=\sqrt{\frac{1}{T}\overset{T}{\underset{t=1}{\sum }}\langle {\left(r_{i}^{\prime }\left(t\right)-r_{i}\left(t_{\text{ref}}\right)\right)}^{2}\rangle ,} \end{array}$$

where *T* is the trajectory time over which the RMSF is calculated, *t*_ref_ is the reference time, *r*_*i*_ is the position of residue *i*, *r*′ is the position of atoms in residue *i* after superposition on the reference, and the angle brackets indicate that the average of the square distance is taken over the selection of atoms in the residue.

PL Contacts: Four categories of interactions (“contacts”) between protein and ligand are hydrophobic, ionic, water bridges, and hydrogen bonds. It allows to examine more specific subtypes in each type of interaction. The stacked bar charts are normalized over the course of the trajectory: For instance, a score of 0.7 indicates that the interaction is maintained for 70% of the total simulation time. Few protein residues could have multiple interactions of the same subtype with the ligand, causing values greater than 1.0 to be feasible.

## 3. Results and Discussion

With the addition of data from the Ayurveda database, alkamid, marine natural products database, and CyanoMetdatabase, COCONUT now has 406,727 distinct natural products. It offers a simple interface and can predict the bioactivity of NPs with a high degree of accuracy. Coumarin substructure NPs were selected due to their antitubercular efficacy. There are 626 NPs in the COCONUT database under the coumarin substructure. LigPrep in the Schrodinger Suite was used to prepare each ligand (NP), and generated 1967 ligands in total. Out of all the ligands, 1761 ligands were docked onto the 4P8N protein. The docking scores were in the range of −11.0852 to 2.9196 kcal/mol. The docking score of the cocrystal ligand (QN118) was −7.676 kcal/mol. Five hundred and thirty-five ligands were having higher docking scores (<−7.676 kcal/mol) than QN118. Ligand–protein interactions are discussed in Table 1. The critical amino acids in the active site region are Gln334, Tyr60, Leu363, and Arg325 [29]. Poor ADME metrics were usually a dealbreaker for molecules in the early stages of drug design. Molecular weight, alogP, HBA, HBD, polar surface area, number of rotatable bonds, number of heavy atoms, aromatic ring counts, and other physicochemical parameters all have an impact on ADMEs. When designing orally active drugs, studies often refer to Lipinski's Ro5 (Lipinski Ro5: Mol.wt 500; alogP 5; HBD 5; HBA 10). If the compound follows this rule, it has a better chance of being bioavailable, or absorbed efficiently by the human body when consumed orally. All the 535 ligands' physicochemical characteristics were calculated. There were 178 ligands that met all the criteria. The binding energy of all 178 ligands was calculated by MMGBSA. The binding energy of the cocrystal ligand QN118 was calculated to be −58.77 kJ/mol. A binding free energy greater than −58.77 kJ/mol has been attained by 12 ligands.

**Figure 2:**
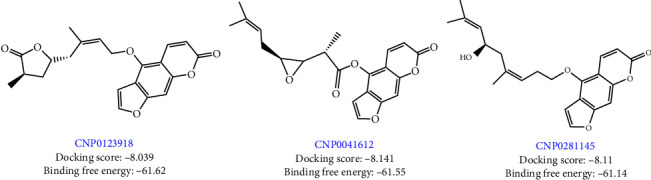
Summary of top three hit molecules.

The chemical structures of the top three hit molecules are shown in Figure 2. In Table 2, the ADME properties of the top three hit compounds are summarized.

Ligand–Protein Interactions in XP Docking: Top hits ligands CNP0123918 (Lucida furanocoumarin B), CNP0041612 (z-Notopterol), and CNP0281145 inside the binding pocket of DprE1 were addressed, and their molecular interactions were compared to those of the cocrystal ligand QN118.

**Figure 3:**
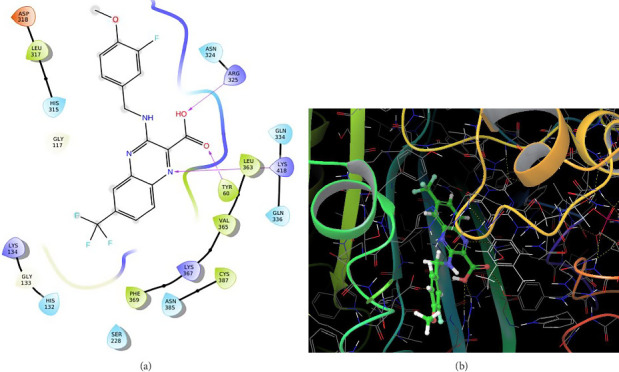
Cocrystal ligand (QN118): (a) 2D interactions with 4P8N; (b) 3D poses with 4P8N.

In QN118, the quinoxaline moiety was settled inside the binding pocket. As shown in Figure 3, residues such as ARG325, LYS418, and TYR60 were involved in the interactions. Quinoline moiety was stabilized inside the binding pocket by forming three hydrogen bonds (i) between ARG325 and −OH of the carboxylic group, (ii) between TYR60 and oxygen of the carboxylic group, and (iii) between LYS418 and nitrogen of the quinoxaline ring.

**Figure 4:**
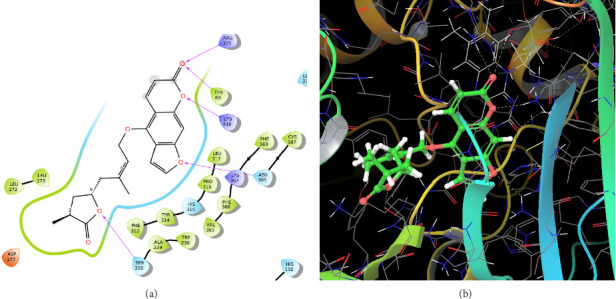
CNP0123918 (lucida furanocoumarin B): (a) 2D interactions with 4P8N; (b) 3D poses with 4P8N.

In CNP0123918, the furanocoumarin moiety is settled inside the active site. As shown in Figure 4, three hydrogen bonds stabilize the ligand, one is from amino acid residue LYS418 to oxygen of coumarin nucleus and the other two are from TYR60 and ARG325 to oxygen attached to the coumarin ring. There is one more hydrogen bond from ASN385 to oxygen nucleus of furano ring attached to coumarin. SER228 also formed hydrogen bond with the oxygen of the substituted furan ring.

**Figure 5:**
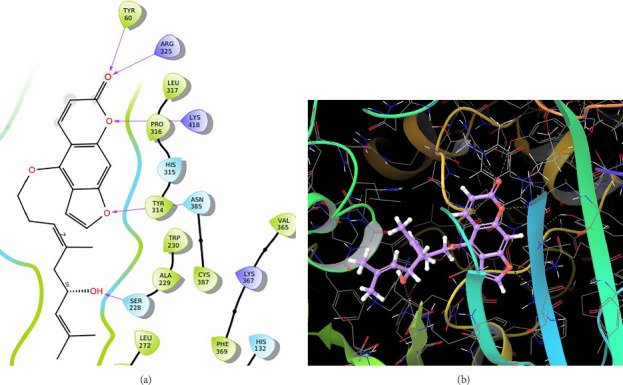
CNP0041612 (z-notopterol): (a) 2D interactions with 4P8N; (b) 3D poses with 4P8N.

In CNP0041612, furanocoumarin moiety is settled inside the binding pocket. As shown in Figure 5, three hydrogen bonds are stabilized by the ligand, one is from amino acid residue LYS418 to oxygen of the coumarin ring, and the other two are from TYR60 and ARG325 to oxygen attached to the coumarin ring. There is one more hydrogen bond from ASN385 to oxygen nucleus of the furano ring attached to coumarin. SER228 also formed hydrogen bond with the hydroxyl group of the substituted chain.

**Figure 6:**
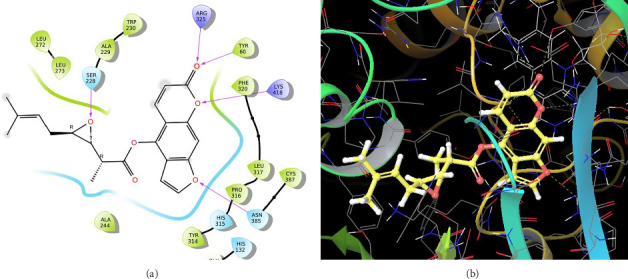
CNP0281145: (a) 2D interactions with 4P8N; (b) 3D poses with 4P8N.

In CNP0281145, furanocoumarin moiety is settled inside the activity site. As shown in Figure 6, three hydrogen bonds stabilize the ligand, one is from amino acid residue LYS418 to oxygen of coumarin nucleus, and the other two are from TYR60 and ARG325 to oxygen attached to the coumarin ring. There is one more hydrogen bond from ASN385 to oxygen nucleus of the furano ring attached to coumarin. SER228 also formed hydrogen bonding with the oxygen of the substituted oxirane ring.

The docked complexes' MMGBSA binding energy values were calculated using the prime module. The cocrystal ligand's (QN118) binding free energy was −58.77 kJ/mol, while the binding free energy (“MMGBSA dG Bind”) for the 178 NPs ranged from −8.1 to −67.42 kJ/mol. Ligand–protein interaction and MMGBSA binding energy were used to narrow down the compounds. The top three hit compounds (Figure 2), CNP0123918, CNP0041612, and CNP0281145, were chosen for MD simulation due to their good binding energies −61.62, −61.55, and −61.14 kJ/mol, respectively.

In molecular dynamics, the adaptability of the whole protein system is considered, and the interactions' dynamic nature under physiological settings was simulated using molecular dynamics. Each of the three hit molecules and the cocrystal ligand underwent a 200-ns dynamics simulation to learn more about the stability and fluctuations of the bound complex.

**Figure 7:**
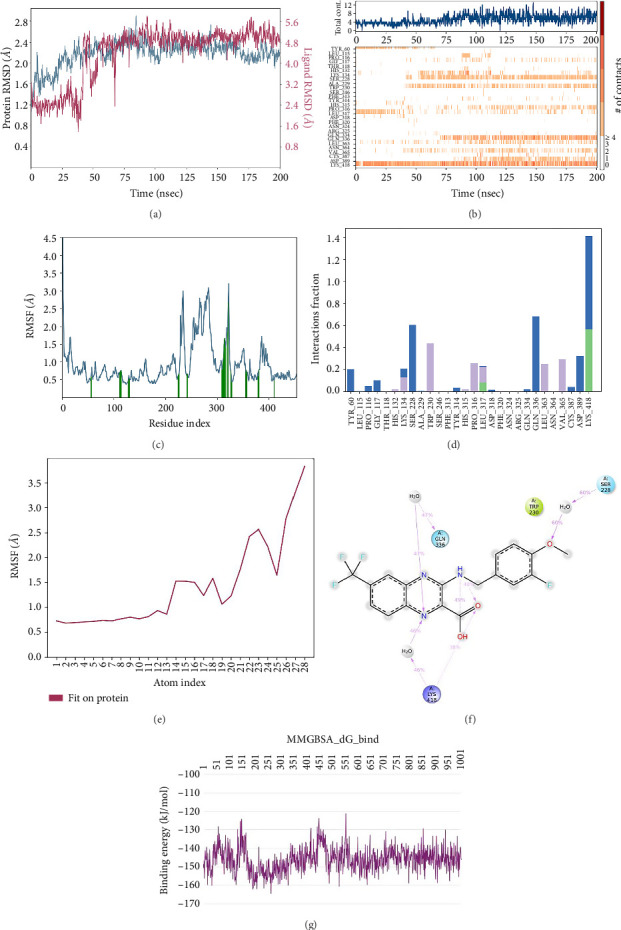
Simulation interaction diagrams of QN118 (cocrystal ligand): (a) RMSD plot of protein–ligand (blue line indicates protein deviation, whereas pink line indicates ligand deviation); (b) protein–ligand timeline contacts; (c) protein RMSF plot; (d) histogram representation of protein–ligand contacts; (e) ligand RMSF plot; (f) protein–ligand interactions in 2D; (g) binding energy throughout the simulation.

In the QN118 (cocrystal ligand) RMSD, the ligand was deviated more between 35 and 50 ns, which later remained stable throughout the simulation. In ligand-RMSD, the deviation was observed between 1.2 and 5.6 Å, whereas in the case of protein, a lower deviation (1.6–2.4 Å) was observed. Mean RMSD of ligand with respect to protein is 4.376 ± 1.059 Å. In the RMSF plot, amino acid residues from 260 to 280 fluctuated above 2.5 Å due to the instability of residues in that region. The oxygen atom of the -COOH group attached to the quinoxaline ring was stabilized by forming a hydrogen bond with LYS418. Two water bridges were formed between the nitrogen atom of the quinoxaline ring and LYS418 and GLN336. One more water bridge existed between the substituted methoxy group oxygen and SER 228. Compared to XP docking, QN118 showed an H-bond with LYS418 and water bridges with LYS418, SER228, and GLN336 but retained hydrogen bonds with TYR60 and ARG325 residues. The PL contacts plot showed a maximum of 10 contacts throughout the simulation. GLN336, SER228, and LYS418 have contributed dominant to the binding of the ligand. After running the simulation, the cocrystal ligand's average binding energy to the protein complex was found to be −145.9065 kJ/mol, with a standard deviation of 6.50 kJ/mol. All the data are shown in Figures 7(a), 7(b), 7(c), 7(d), 7(e), 7(f), and 7(g).

**Figure 8:**
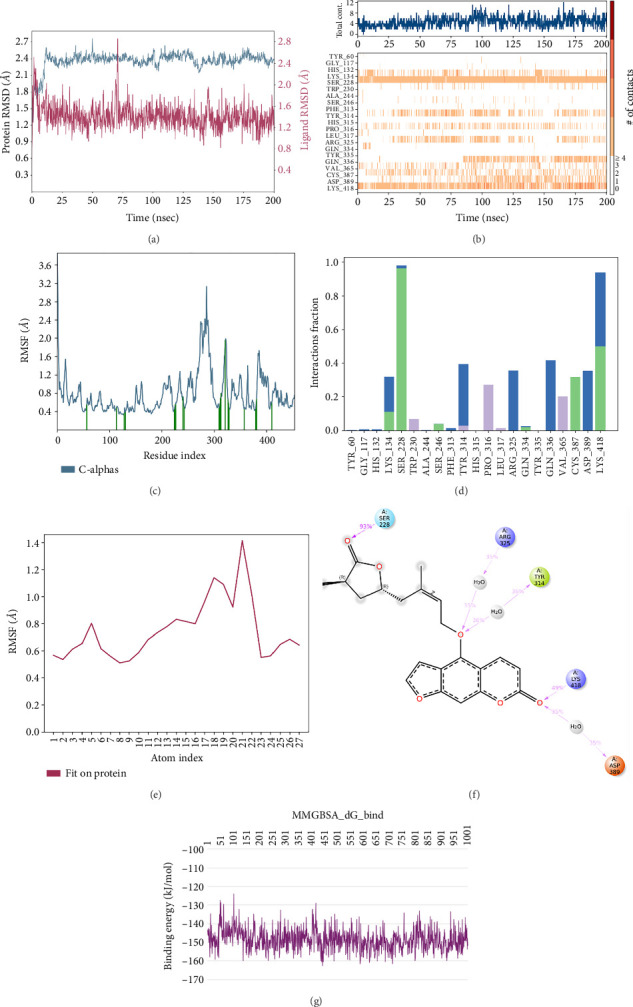
Simulation interaction diagrams of CNP0123918: (a) RMSD plot of protein–ligand (blue line indicates protein deviation, whereas pink line indicates ligand deviation); (b) protein–ligand timeline contacts; (c) protein RMSF plot; (d) histogram representation of protein–ligand contacts; (e) ligand RMSF plot; (f) protein–ligand interactions in 2D; (g) binding energy throughout the simulation.

In CNP0123918 RMSD, the ligand was slightly deviated above 2.7 Å at 75 ns, which later remained stable throughout the simulation. In ligand-RMSD, the deviation was observed between 1.2-2.0 Å, whereas in the case of protein, a higher deviation (1.8–2.4 Å) was observed. Mean RMSD of ligand with respect to protein is 1.409 ± 0.22 Å. In the RMSF plot, amino acid residues from 270 to 280 fluctuated above 2.4 Å due to the instability of residues in that region. The coumarin ring was stabilized by a hydrogen bond and a water bridge between the oxygen (C=O) and LYS418 and ASP389, respectively. One more hydrogen bond existed between the furan ring oxygen and SER 228. There were two water bridges between the oxygen attached to furanocoumarin and ARG325 and TYR314. Compared to XP docking, CNP0123918 has shown an H-bond with LYS418 and water bridges with TYR314 and ASP389 but retained hydrogen bonds with TYR60 and ASN385 residues. The PL contacts plot showed a maximum of 10 contacts throughout the simulation. LYS134, SER228, TYR314, ARG325, ASP389, and LYS418 have contributed dominant to the binding of the ligand. After running the simulation, the CNP0123918's average binding energy to the protein complex was found to be −148.4766 kJ/mol, with a standard deviation of 5.66 kJ/mol. All the data are shown in Figures 8(a), 8(b), 8(c), 8(d), 8(e), 8(f), and 8(g).

**Figure 9:**
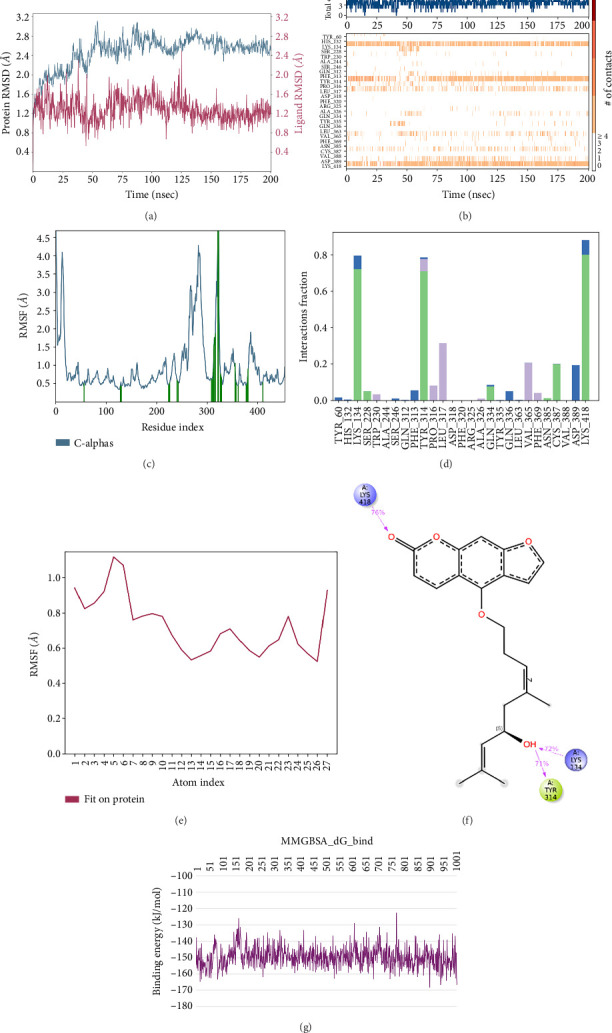
Simulation interaction diagrams of CNP0041612: (a) RMSD plot of protein–ligand (blue line indicates protein deviation, whereas pink line indicates ligand deviation); (b) protein–ligand timeline contacts; (c) protein RMSF plot; (d) histogram representation of protein–ligand contacts; (e) ligand RMSF plot; (f) protein–ligand interactions in 2D; (g) binding energy throughout the simulation.

In CNP0041612 RMSD, the ligand was stable throughout the simulation. In ligand-RMSD, the deviation was observed between 1.2 and 2.0 Å, whereas in the case of protein, a higher deviation (2.0–2.8 Å) was observed. Mean RMSD of ligand with respect to protein is 1.285 ± 0.231 Å. In the RMSF plot, amino acid residue 17 fluctuated above 4.0 Å, and residues 264 to 288 fluctuated between 3.0 and 4.5 Å due to the instability of residues in that region. The coumarin ring was stabilized by a hydrogen bond between the oxygen (C=O) and LYS418. Two more hydrogen bonds existed between the substituted hydroxyl group and LYS134 and TYR314. Compared to XP docking, CNP041612 has shown an H-bond with LYS134 and TYR314 but retained hydrogen bonds with TYR60, ARG325, SER228, and ASN385 residues. The PL contacts' plot showed a maximum of seven contacts throughout the simulation. LYS134, TYR314, and LYS418 have contributed dominant to the binding of the ligand. After running the simulation, the CNP0041612's average binding energy to the protein complex was found to be −147.7094 kJ/mol, with a standard deviation of 6.07 kJ/mol. All the data are shown in Figures 9(a), 9(b), 9(c), 9(d), 9(e), 9(f), and 9(g).

**Figure 10:**
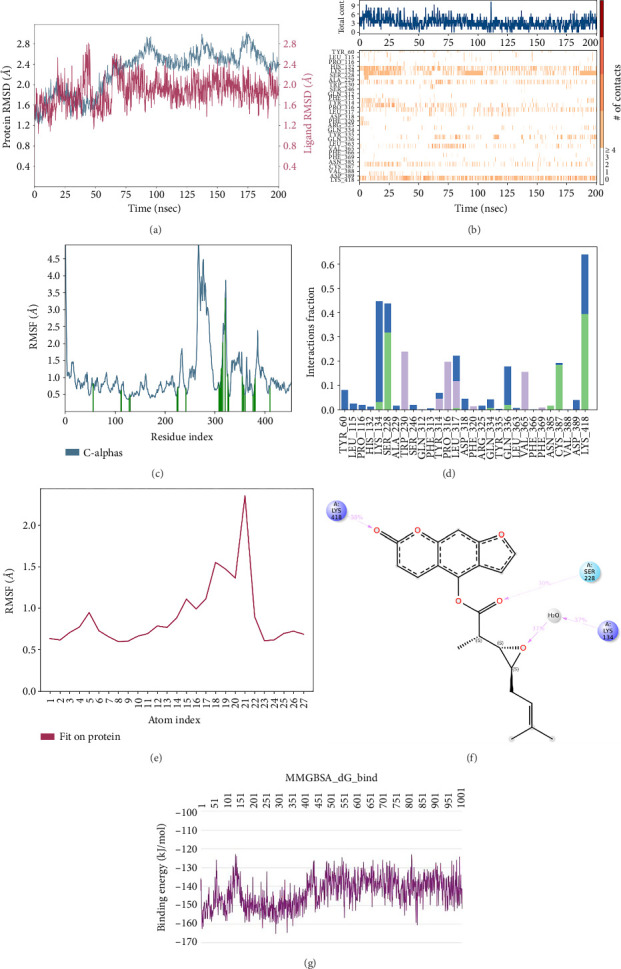
Simulation interaction diagrams of CNP0281145: (a) RMSD plot of protein–ligand (blue line indicates protein deviation, whereas pink line indicates ligand deviation); (b) protein–ligand timeline contacts; (c) protein RMSF plot; (d) histogram representation of protein–ligand contacts; (e) ligand RMSF plot; (f) protein–ligand interactions in 2D; (g) binding energy throughout the simulation.

In CNP0281145 RMSD, the ligand was deviated above 2.7 Å between 35 and –50 ns, which later remained stable throughout the simulation. The deviation was observed between 1.6 and 2.8 Å for both protein and ligand. Mean RMSD of ligand with respect to protein is 1.866 ± 0.278 Å. In the RMSF plot, amino acid residues 267 to 280 fluctuated above 4.5 Å due to the instability of residues in that region. The coumarin ring was stabilized by a hydrogen bond between the oxygen (C=O) and LYS418. One more hydrogen bond existed between the substituted oxygen atom (C=O) and SER 228. There was a water bridge between the oxygen (three-membered ring) and LYS134. Compared to XP docking, CNP0281145 has shown H-bonds with LYS418 and SER228 and a water bridge with LYS134 but retained hydrogen bonds with ARG325, TYR60, and ASN385 residues. The PL contacts' plot showed a maximum of nine contacts throughout the simulation. LYS134, SER228, and LYS418 have contributed dominant to the binding of the ligand. After running the simulation, the CNP0231145's average binding energy to the protein complex was found to be −143.1253 kJ/mol, with a standard deviation of 8.05 kJ/mol. All the data are shown in Figures 10(a), 10(b), 10(c), 10(d), 10(e), 10(f), and 10(g).

Some docking-predicted molecular interactions were preserved following MD simulation, whereas new interactions were discovered that had not been predicted by XP docking (Table 3). The 200-ns simulations showed that CNP0123918 had 93% of interaction with SER228, the best stability, and the highest number of interactions. There was no significant change in the structure of the complex (RMSD > 2.4 Å) during the simulation with a maximum of eight interactions. The coumarin ring containing carbonyl oxygen is responsible for stability by forming hydrogen bonds with LYS418. Whereas CNP0041612 and CNP0281145 had 76% interaction with LYS418, 72% interaction with LYS314, and 38% interaction with LYS418, respectively. QN118, the cocrystal ligand, was reported as a DprE1 inhibitor [30] and had only 38% interaction with the LYS418 residue and a low binding energy of −58.77 kJ/mol compared to the hit molecules. And standard TB drugs such as ethambutol (EMB) and pyrazinamide (PYR) had docking scores of −2.183 and −5.231 kcal/mol and binding energies of −27.16 and −22.63 kJ/mol, respectively. These standard drugs were showing good results in vitro and in vivo evaluation but did not perform well in silico studies. The three hit molecules contain furanocoumarin, a tricyclic aromatic compound (fused furan ring to coumarin) mainly found in higher plants such as citrus plants (grapefruits) [31]. Furanocoumarins were reported to have antitubercular efficacy against the *MTB* reference strain H37Rv [32]. In comparison to the cocrystal ligand and standard TB drugs, these three hit compounds demonstrated strong activity against DprE1 in silico studies.

## 4. Conclusion

The 626 compounds in the natural products' database were screened using Schrodinger Maestro. Three compounds were shown to be effective inhibitors of DprE1 after further sorting by XP docking and MMGBSA screening. The binding contacts of the top three hits are moderate to strong within the active region of DprE1, as determined by molecular docking studies. To further highlight the dynamic nature and stability of interactions within the active site, MD simulations of 200 ns were performed on these three molecules. Key interactions with amino acid residues, including LYS418 and SER228, were shown to be necessary for DprE1 inhibition, as revealed by both MD and docking studies. Docking scores and binding free energies for CNP0041612 and CNP0281145 were good, but they showed fewer interactions with amino acid residues throughout the simulation compared to CNP0123918. Future research will focus on the experimental validation of CNP0123908 to establish its potential as an effective DprE1 inhibitor. This will be undertaken through a two-pronged strategy involving in vitro enzymatic assays (determining IC_50_ values and cytotoxicity in mammalian cell lines) and in vivo efficacy (pharmacokinetics and bioavailability, efficacy in murine TB models, and toxicological assessment).

## Figures and Tables

**Table 1:** Docking score and MMGBSA results of top 12 molecules.

Ligand	Interaction with an amino acid		Docking score (kcal/mol)	MMGBSA dG bind (kJ/mol)
	H-bond	Pi-pi/pi-cat		
CNP0123918	LYS418, SER228, TYR60, ARG325	—	−8.039	−61.62
CNP0041612	LYS418, SER228, TYR60, ARG325	—	−8.141	−61.55
CNP0281145	LYS418, SER228, TYR60, ARG325	—	−8.11	−61.14
CNP0395543	SER228	PHE320, TRP230	−7.788	−61.13
CNP0313439	LEU317, ASP318	—	−7.758	−61.03
CNP0387429	SER228	—	−8.183	−60.87
CNP0028115	LYS418, ASN385	—	−7.902	−60.08
CNP0167622	ARG325	—	−7.83	−59.62
CNP0234596	—	PHE320	−8.134	−59.53
CNP0209139	SER228	PHE320, TRP230	−8.397	−59.46
CNP0141083	SER228, LEU317	—	−8.238	−59.28
CNP0123617	LYS418, SER228	—	−7.785	−59.24
QN118 (co-crystal ligand)	ARG325, LYS418, TYR60	—	−7.676	−58.77

**Table 2:** Physicochemical properties of the top three molecules.

Ligands	Docking score (kcal/mol)	MMGBSA dG bind (kJ/mol)	Mol.wt	alogP	HBA	HBD	PSA	Rot bonds	QlogK_p_	Ro5 violations	%Human oral absorption
CNP0123918	−8.039	−61.62	368.379	3.892	6	0	78.88	5	−2.561	0	92.91
CNP0041612	−8.141	−61.55	368.423	4.582	5	1	72.81	7	−1.679	0	100
CNP0281145	−8.11	−61.14	368.379	3.965	6	0	82.18	6	−1.867	0	100

**Table 3:** Comparison of interactions between XP docking and MD simulation.

Ligand	XP docking	MD simulation		Binding energy (kJ/mol)
	H-bond	H-bond	Water bridge	
CNP0123918	ARG325, TYR60, LYS418, ASN385, SER228	LYS418 (49%), SER228 (93%)	TYR314 (36%), ARG325 (35%), ASP389 (35%)	−148.4766
CNP0041612	ARG325, TYR60, LYS418, ASN385, SER228	LYS418 (76%), LYS134 (72%), TYR314 (71%)	—	−147.7094
CNP0281145	ARG325, TYR60, LYS418, ASN385, SER228	LYS418 (38%), SER228 (30%)	LYS134 (37%)	−143.1253
QN118	TYR60, LYS418, ARG325	LYS418 (38%)	SER228 (60%), LYS418 (46%), GLN336 (47%)	−145.9065

## Data Availability

The data that support the findings of this study are available from the corresponding author upon reasonable request.
